# Elastic and collapsible: current understanding of cell walls in succulent plants

**DOI:** 10.1093/jxb/erac054

**Published:** 2022-02-15

**Authors:** Marc Fradera-Soler, Olwen M Grace, Bodil Jørgensen, Jozef Mravec

**Affiliations:** 1 Department of Plant and Environmental Sciences, University of Copenhagen, Thorvaldsensvej 40, 1871 Frederiksberg, Denmark; 2 Royal Botanic Gardens, Kew, Richmond, Surrey, UK; 3 University of Cambridge, UK

**Keywords:** Cell wall composition, cell wall folding, cell wall remodelling, collapsible cell walls, drought avoidance, plant cell walls, plant glycomics, polysaccharides, succulent plants

## Abstract

Succulent plants represent a large functional group of drought-resistant plants that store water in specialized tissues. Several co-adaptive traits accompany this water-storage capacity to constitute the succulent syndrome. A widely reported anatomical adaptation of cell walls in succulent tissues allows them to fold in a regular fashion during extended drought, thus preventing irreversible damage and permitting reversible volume changes. Although ongoing research on crop and model species continuously reports the importance of cell walls and their dynamics in drought resistance, the cell walls of succulent plants have received relatively little attention to date, despite the potential of succulents as natural capital to mitigate the effects of climate change. In this review, we summarize current knowledge of cell walls in drought-avoiding succulents and their effects on tissue biomechanics, water relations, and photosynthesis. We also highlight the existing knowledge gaps and propose a hypothetical model for regulated cell wall folding in succulent tissues upon dehydration. Future perspectives of methodological development in succulent cell wall characterization, including the latest technological advances in molecular and imaging techniques, are also presented.

## Teaser

The mechanistic adaptations that allow the cell walls of succulent plants to fold provide insights into cellular water storage and the reversible responses to drought and rehydration.

## Introduction

**Fig. 1.**
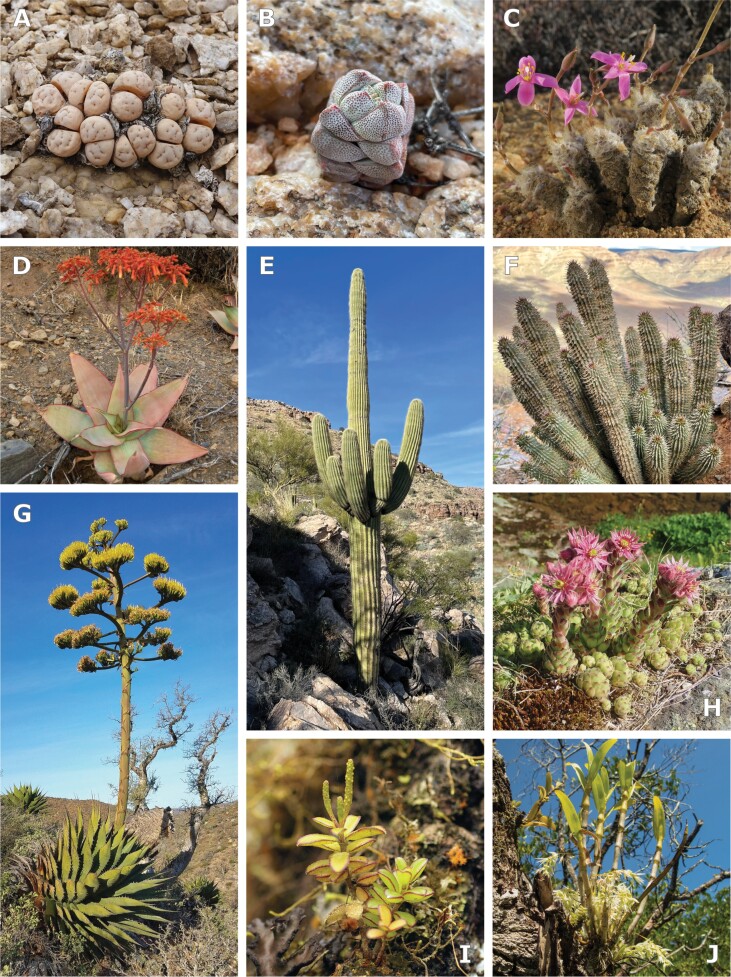
Succulence can occur in any plant organ, with leaf succulents and stem succulents being the most familiar. (A–G) Examples of drought-avoiding succulent plants from arid and semi-arid regions of the world. (A) *Lithops ruschiorum* (Aizoaceae) (photo: John Barkla; https://www.inaturalist.org/observations/3179166). (B) *Crassula deceptor* (Crassulaceae) (photo: Matt Berger; https://www.inaturalist.org/observations/96923687). (C) *Anacampseros filamentosa* (Anacampserotaceae) (photo: Kevin Murray; https://www.inaturalist.org/observations/18098778). (D) *Aloe striata* (Asphodelaceae) (photo: Christiaan Viljoen; https://www.inaturalist.org/observations/91416316). (E) *Carnegiea gigantea* (Cactaceae) (photo: Matt Berger; https://www.inaturalist.org/observations/105300210). (F) *Hoodia gordonii* (Asclepiadoideae, Apocynaceae) (photo: Matt Berger; https://www.inaturalist.org/observations/97449791). (G) *Agave shawii* (Asparagaceae) (photo: Alan Rockefeller; https://www.inaturalist.org/observations/21007526). (H–J) Examples of drought-avoiding succulent plants from xeric microhabitats. (H) *Sempervivum montanum* (Crassulaceae) (photo: Julien Renoult; https://www.inaturalist.org/observations/6840361). (I) *Peperomia galapagensis* (Piperaceae) (photo: Anja Junghanns; https://www.inaturalist.org/observations/70609760). (J) *Dendrobium kratense* (Orchidaceae) (photo: Gerard Chartier; https://www.inaturalist.org/observations/63818588). All photos from iNaturalist. (A, H) Licensed under CC1.0; (B–F, I, J) licensed under CC-BY-4.0; (G) licensed under CC-BY-SA.

With their peculiar appearance and their capacity to thrive under extreme conditions, succulent plants have long captivated botanists and plant enthusiasts (Eggli, 2017). Drought-avoiding succulent plants store water in living cells for later remobilization, which renders them temporarily independent of an external water supply (see Box 1) (Eggli and Nyffeler, 2009; Griffith and Males, 2017). Water-storage capacity in succulents is usually accompanied by several co-adaptive traits, such as certain xeromorphic features and different degrees of crassulacean acid metabolism (CAM), so that succulence emerges as a complex adaptive syndrome (Ogburn and Edwards, 2010; Winter *et al.*, 2015; Males, 2017). The link between succulence and CAM is an ongoing debate: succulence has long been regarded as a prerequisite for CAM, and succulence and strong CAM are highly correlated (Kluge and Ting, 1978; Sayed, 2001; Lüttge, 2004), but it remains unclear whether the co-occurrence of CAM and succulence is due to mutual facilitation or just a result of co-selection under similar selective pressures (Ogburn and Edwards, 2010; Heyduk *et al.*, 2016; Edwards, 2019; Leverett *et al.*, 2021). Succulence and its co-adaptive traits have evolved in numerous lineages across the plant tree of life (Fig. 1) (Nyffeler and Eggli, 2010; Edwards, 2019). Among photosynthetic succulent organs, a widely used functional classification, coined by Ihlenfeldt (1985), considers two types of succulence: all-cell succulence (e.g. Crassulaceae; Fradera-Soler *et al.*, 2021), with all cells both performing photosynthesis and storing water, and storage succulence (e.g. *Aloe*, Asphodelaceae; Ni *et al.*, 2004*a*), in which there is a functional demarcation between photosynthetic tissue (i.e. chlorenchyma) and water-storing tissue (i.e. hydrenchyma). In reality, the anatomical diversity of succulent organs is even larger when considering the intermediate states between all-cell and storage succulence and the various arrangements of hydrenchyma and chlorenchyma within an organ. The term ‘succulent tissue’ is usually applied to those tissues in succulent organs responsible for water storage, which are constituted primarily of highly vacuolated parenchyma cells with thin, elastic primary cell walls (Kluge and Ting, 1978; Gibson and Nobel, 1986; von Willert *et al.*, 1992). Thus, ‘succulent tissue’ may refer specifically to the hydrenchyma in a storage succulent or to all parenchyma cells in an all-cell succulent organ.

**Box 1. Ecology of succulents**

Drought can lead to different degrees of water stress in plants, defined as ‘situations in which plant water potential and turgor are reduced enough to interfere with normal functioning’, although the ‘exact cell water potential at which this occurs depends on the kind of plant’ (Kramer, 1983). Many drought-resistant plants (as defined by Levitt, 1980) are drought tolerant and are able to track soil water potential to exceptionally low values (Walter and Stadelmann, 1974; Pockman and Sperry, 2000; Griffiths and Males, 2017); this category includes ‘true’ xerophytes and the extreme case of resurrection plants, which are additionally desiccation tolerant. However, most succulent plants do not tolerate low water potentials (Ψ) and are therefore regarded as drought avoiders, with stored water delaying or completely preventing the effects of water stress at the cellular/tissue level (Eggli and Nyffeler, 2009; Ogburn and Edwards, 2010); this review focuses on drought-avoiding succulents. Succulence may be linked to other ecological strategies, most notably halophytism (Kadereit *et al.*, 2003; Flowers and Colmer, 2008), although halophytic succulents are functionally distinct from drought-avoiding succulents.

Despite being traditionally associated with arid and hyper-arid deserts (‘true’ deserts as defined by Laity, 2008), drought-avoiding succulents need to refill their water stores periodically and are therefore dependent on seasonally predictable rainfall, typical of semi-arid habitats (von Willert *et al.*, 1992). Thus, the hotspots of succulent diversity tend to occur in semi-arid habitats and desert fringes (Burgess and Shmida, 1988; Ogburn and Edwards, 2010). Drought-avoiding succulents are also well represented in xeric microhabitats within relatively hydric habitats (Fig. 1H–J), as is the case with many epiphytes (Zotz, 2016) and plants in some alpine niches (Körner, 2003).

**Fig. 2.**
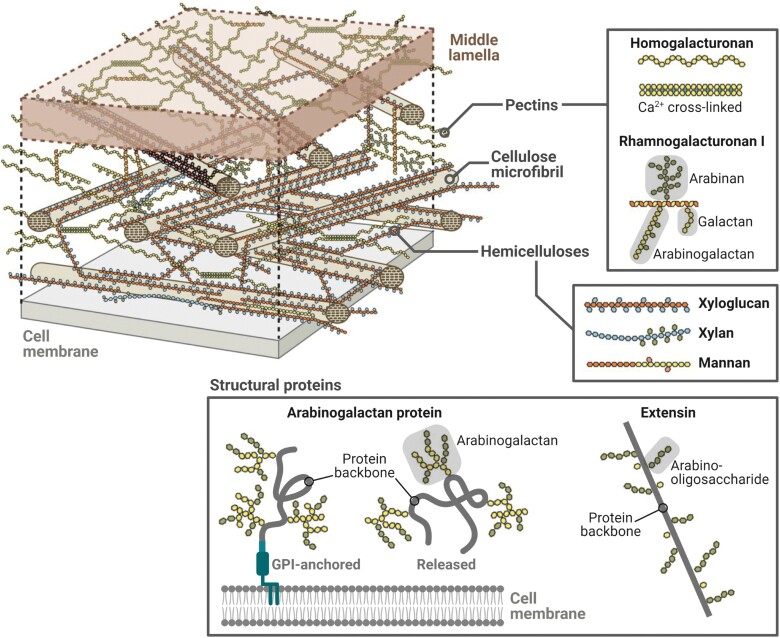
Three-dimensional molecular model of type I primary cell wall typical of most angiosperms (except the commelinids), showing the molecular interactions between the cell wall polysaccharides. The boxes show some representatives of the two groups of non-cellulosic cell wall polysaccharides and of cell wall structural proteins (not included in the three-dimensional model). Modified from Carpita *et al.* (2015). The cell wall. In: Buchanan BB, Gruissem W, Jones RL, eds. Biochemistry and Molecular Biology of Plants. 2nd edition. 45–110. © 2015 John Wiley and Sons, Ltd. Created with BioRender.com.

Across the plant tree of life, variation in cell wall structure and composition governs plant morphology and physiology and has undoubtedly played a crucial role in the adaptation to different evolutionary pressures (Sarkar *et al.*, 2009; Sørensen *et al.*, 2010). Primary cell walls are complex and dynamic systems capable of deformation due their intrinsic viscoelasticity (Niklas, 1992; Braybrook *et al.*, 2012; Cosgrove, 2018). They are composed of three coextensive polymeric networks: (i) a tension-bearing cellulose-hemicellulose network, (ii) a water-retentive, gel-forming pectin network, and (iii) a structural protein network (Fig. 2) (Cosgrove, 2005; Albersheim *et al.*, 2011; Carpita *et al.*, 2015). Hemicelluloses, pectins, and structural proteins are highly diverse, and differing abundances and arrangements of these components result in contrasting cell wall characteristics (Showalter, 1993; Willats *et al.*, 2001; Harholt *et al.*, 2010; Scheller and Ulvskov, 2010). These characteristics can be modified through cell wall remodelling, which affects cell wall structure and/or composition (see Box 2). Some cell wall polysaccharides, known as cell wall storage polysaccharides (CWSPs), appear to have been evolutionarily repurposed for storage and other functions across several plant lineages (see Box 3).

**Box 2. Cell wall remodelling**

The primary cell wall is a dynamic system whose properties can be tightly controlled via cell wall remodelling, which involves controlled modification, rearrangement, degradation, and/or reconstruction of the cell wall in both growing and mature cells in response to various stimuli (Barnes and Anderson, 2018; Anderson and Kieber, 2020). Cell wall extension and contraction are generally regarded as a consequence of cell wall remodelling through the processes of cell wall loosening (i.e. cell wall stress relaxation and increased extensibility) and/or softening (i.e. reduced stiffness and increased deformability; Cosgrove, 2018; Zhang *et al.*, 2019). Cell wall loosening is thought to be mediated by expansins, a class of non-enzymatic proteins that weaken non-covalent bonds in the cellulose–hemicellulose network and allow for slippage among cell wall components, whereas the activity of several hemicellulose- and pectin-modifying enzymes can lead to cell wall softening and secondary loosening (Cosgrove, 2016, 2018). These enzymes comprise xyloglucan endo-transglycosylases/hydrolases (XTHs), pectin methylesterases (PMEs), pectin acetylesterases (PAEs), polygalacturonases (PGs), and pectate lyases (PLs), among others (Eklöf and Brumer, 2010; Sénéchal *et al.*, 2014). There has been a growing interest in cell wall remodelling in response to abiotic stress due to its potential applications in near-future climate change scenarios (e.g. Le Gall *et al.*, 2015; Tenhaken, 2015; Ezquer *et al.*, 2020). A large proportion of plant genes are involved in cell wall synthesis, assembly and remodelling (~15% of the genome in *Arabidopsis*; Arabidopsis Genome Initiative, 2000; Carpita *et al.*, 2001), and shifts in the expression patterns of these genes in response to different stresses have been widely reported (Houston *et al.*, 2016), which highlights the relevance of cell walls in the stress response.

**Box 3. Cell wall storage polysaccharides**

Cell wall storage polysaccharides (CWSPs) are apoplastic polysaccharides associated with the cell wall that can be repurposed for energy storage and other functions (Meier and Reid, 1982). They comprise mannans, xyloglucans, and (arabino)galactans, and are mobilized from the cell wall via various enzymatic activities (Buckeridge *et al.*, 2000; Buckeridge, 2010). In many cases, CWSPs occur as a special deposition inside the ordinary primary cell wall. Among mannan CWSPs, insoluble ‘pure’ mannans have been linked to increased hardness and are abundant in seeds, whereas soluble mannans, formed by substitution with galactosyl residues [i.e. galacto(gluco)mannans] and/or acetylation, have been reported in succulent-like storage organs, such as orchid pseudobulbs and underground organs of geophytes, where they are believed to play a role in cellular water relations and water storage (Stancato *et al.*, 2001; Wang *et al.*, 2006; Ranwala and Miller, 2008; Chua *et al.*, 2013).

Cell wall properties are expected to be decisive in overcoming the alleged biomechanical and physiological challenges posed by the succulent syndrome. Besides being involved in mechanical support, cell walls in succulent tissues are capable of folding, which allows for reversible changes in the volume of succulent organs during dehydration/rehydration cycles while preventing catastrophic cell collapse and irreversible damage (von Willert *et al.*, 1992; Christensen-Dean *et al.*, 1993; Mauseth, 1995; Burgoyne *et al.*, 2000; Bobich and North, 2009). Secondly, cell walls are the gas–liquid interface in the parallel processes of CO_2_ diffusion and water movement in photosynthetic organs, thus influencing the interplay of factors linked to limitation of photosynthesis (Barbour, 2017; Gago *et al.*, 2019). Therefore, water relations and CO_2_ uptake in succulents are expected to be tightly controlled by cell wall characteristics (Flexas *et al.*, 2013; Xiong *et al.*, 2017; Xiong and Nadal, 2020). Despite the general assumption that cell wall characteristics play a pivotal role in the succulent syndrome, the cell walls of succulent plants have received little research attention to date. Studies have been hampered by the challenges of applying standard histological and biochemical techniques to water-rich tissues, with methodological modifications often being required in order to investigate succulent tissues (e.g. Ahl *et al.*, 2018; Mozzi *et al.*, 2021).

Increasing surface temperature and expanding aridity in many parts of the world (Intergovernmental Panel on Climate Change, 2007) are intensifying the need for deeper insights into the mechanisms of drought resistance and water management in plants. CAM-performing succulent plants have been identified as natural capital to mitigate the effects of climate change (Grace, 2019), including the possibility of engineering CAM into crops (Borland *et al.*, 2014; Yang *et al.*, 2015). However, while several succulence-related traits will probably allow many succulent groups to better withstand future climatic conditions (Willis, 2017), other succulent taxa are facing a high risk of extinction (Goettsch *et al.*, 2015; Guo *et al.*, 2016; Young *et al.*, 2016). A better understanding of the mechanisms underlying the succulent function would reaffirm the role of succulent plants as natural capital and would help to promote conservation efforts. This review focuses on the current knowledge of cell walls in drought-avoiding succulent plants and their influence on the function of the succulent syndrome, and highlights the knowledge gaps in these topics. Future perspectives of the characterization of cell walls in succulents and its challenges are also presented.

## Functional relations between cell wall components and responses to drought

Cell wall responses to drought and other abiotic stresses, most of which involve differential gene expression leading to cell wall remodelling, have been widely studied and reviewed in crop and model plants (Le Gall *et al.*, 2015; Tenhaken, 2015; Ezquer *et al.*, 2020). These acclimation processes highlight the importance of cell walls in drought resistance and can also hint at cell wall adaptations in succulents that may have shaped their evolution into drought-prone habitats.

Since the highly labile pectin network strongly influences many interrelated cell wall properties (e.g. thickness, porosity, hydration, elasticity), changes in pectin are likely crucial to drought-induced cell wall remodelling (Harholt *et al.*, 2010; Braybrook *et al.*, 2012; Levesque-Tremblay *et al.*, 2015; Bidhendi and Geitmann, 2016). The nature of pectin gels is determined, at least partially, by the degree of methyl-esterification (DM) of homogalacturonans (HGs), which is regulated by pectin methylesterases, resulting in the formation of either ‘strong’ gels that stiffen the cell wall or ‘weak’ gels that soften it (Hocq *et al.*, 2017). Other pectin-modifying enzymes, such as pectin acetylesterases, polygalacturonases, and pectate lyases, also influence the properties of the pectin matrix. Xyloglucan, the most abundant hemicellulose in primary cell walls of spermatophytes, is targeted by xyloglucan endo-transglycosylases/hydrolases, which can perform two different catalytic activities and either strengthen or soften the cell wall (Eklöf and Brumer, 2010; Scheller and Ulvskov, 2010; Nishikubo *et al.*, 2011). Contrasting patterns of regulation in response to drought have been reported among pectin- and xyloglucan-modifying enzymes (Pelloux *et al.*, 2007; He *et al.*, 2009; Clauw *et al.*, 2015; Nguyen *et al.*, 2016; Xuan *et al.*, 2016), which highlights the complex relationship between these enzymatic activities and cell wall properties. On the other hand, drought stress has been strongly linked to the up-regulation of a large portion of expansin isoforms (Harb *et al.*, 2010; Chen et al., 2019, 2020; Jin *et al.*, 2020), which suggests that adjustments of cell wall loosening and extensibility are general responses against drought.

Pectin gel properties are also determined by rhamnogalacturonan I (RG-I), whose side chains influence cell wall hydration and elasticity (Willats *et al.*, 2001; Harholt *et al.*, 2010). Drought stress has been associated with an increase in the amount of arabinan, galactan, and arabinogalactan RG-I side chains (Leucci *et al.*, 2008; Gribaa *et al.*, 2013). Due to the high mobility of RG-I arabinans and galactans in the cell wall, they have been postulated as cell wall plasticizers, which maintain the fluidity of the pectin network and stabilize the cell wall during dehydration and rehydration (Harholt *et al.*, 2010). This is a particularly relevant feature for cells that undergo drastic changes in shape as water is lost during drought. Structurally highly complex rhamnogalacturonan II (RG-II) side chains, which are thought to provide mechanical strength to the cell wall by forming borate cross-links (O’Neill *et al.*, 2004), also seem to increase in number in response to drought stress (Leucci *et al.*, 2008), although the interpretation of this response is not as clear because the exact physiological role of RG-II is still relatively unknown.

Drought stress has also been associated with the up-regulation of arabinogalactan proteins (AGPs) (Cui *et al.*, 2012; Mareri *et al.*, 2019). Periplasmic AGPs, many of which are anchored to the plasma membrane, seem to occur in a reticulate pattern along the external face of the cell membrane, where they help to maintain the membrane–cell wall continuum by interacting with cell wall components (Gens *et al.*, 2000; Liu *et al.*, 2015). Given that this continuum can be compromised during abiotic stress, the up-regulated AGPs are believed to form a ‘buffer zone’ that stabilizes the membrane by preventing its direct interaction with the cell wall (Lamport *et al.*, 2006). Indeed, a decrease in AGP epitopes and their rearrangement have been linked to the disruption of the membrane–wall continuum in senescing fruits (Leszczuk *et al.*, 2020). AGPs have also been postulated as cell wall plasticizers (Lamport *et al.*, 2006) and may perform a similar role to that of the aforementioned RG-I side chains during dehydration. Another type of cell wall structural protein, extensins, are generally thought to form self-assembling scaffolds that strengthen the wall (Cannon *et al.*, 2008). However, gene expression studies have given contrasting results regarding the regulation of different extensin genes upon drought (Molina *et al.*, 2008; Cevher-Keskin, 2019), which suggests that different extensin isoforms may be performing different functions in the cell wall. Several functions of cell wall structural proteins and their involvement in the drought response remain largely hypothetical, which presents many research opportunities.

## Structure and function of cell walls in succulents

### Biomechanics

Succulent organs tend to have a low surface area to volume ratio to minimize water loss and enhance water storage (Males, 2017), but the considerable weight of stored water poses a biomechanical problem. Cell walls in succulent organs are thus expected to have inherent mechanical properties allowing for efficient mechanical support. Small globose or prostrate succulent plants possess succulent organs that mostly lack support tissues, which is the case for the leaves of Aizoaceae, Crassulaceae, and succulent Asteraceae, and the stems of small members of Cactaceae and some succulent Asteraceae and Asclepiadoideae (Apocynaceae; Gibson, 1996; Ogburn and Edwards, 2010). High cell turgor pressure in these succulent organs generates high hydrostatic pressure and provides most of the mechanical support (Niklas, 1992; Gibson, 1996; Bobich and North, 2009), which also makes them capable of drastic shrinking upon drought (Mauseth, 2006). As a remarkable exception, despite their relatively large size, succulent leaves of *Aloe* and closely related genera lack support tissues and are also primarily supported by hydrostatic pressure on a reinforced epidermis (Gibson, 1996).

Most large succulent organs usually possess support tissues, such as hypodermis, fibres, and wood and bark from secondary growth (Blunden, 1973; Koller and Rost, 1988*a*; Mauseth, 2004*a*, *b*, 2006). There has been a growing interest in the support tissues and their cell walls in certain succulent lineages due to their adaptive and evolutionary relevance or their useful applications, such as the different types of wood of Cactaceae (Vázquez-Sánchez *et al.*, 2017; Reyes-Rivera *et al.*, 2018; Maceda *et al.*, 2019) and the sclerenchyma fibres of *Agave* (Asparagaceae; Ferreira *et al.*, 2014; Hidalgo-Reyes *et al.*, 2015). Despite having support tissues, most large succulent plants are still capable of a high degree of volume change, which may be facilitated by morphological adaptations such as ribs in many Cactaceae and succulent Apocynaceae and Euphorbiaceae (Gibson and Nobel, 1986; Nobel, 1988; Felger and Henrickson, 1997; Eggli and Giorgetta, 2020). Most succulents undergo successive cycles of dehydration and rehydration following external water availability, which is reflected in shrinking and swelling of their succulent organs as the water stores are emptied and refilled (Gibson and Nobel, 1986; von Willert *et al.*, 1992). Even in large succulents with support tissues, turgor pressure still plays an important role in mechanical support compared with non-succulent plants (Schulte *et al.*, 1989; Bobich and North, 2009).

Since drastic changes in the volume of succulent organs can compromise tissue function, succulent taxa capable of extreme shrinking often exhibit secondary cell wall thickenings, which provide structural support during dehydration and restrict the direction of shrinkage of cells. In the notoriously drought-resistant genus *Sansevieria* (syn. *Dracaena*, Asparagaceae), many species exhibit secondary cell wall bands in the hydrenchyma (Koller and Rost, 1988*a*, *b*). Similarly, wide-band tracheids occur in the vascular tissues of succulent organs in many genera of succulent families of the Caryophyllales, namely Cactaceae, Aizoaceae, Anacampserotaceae, and Didiereaceae; these tracheids have annular or helical secondary wall thickenings that extend deeply into the lumen (Landrum, 2001, 2006; Mauseth, 2004*c*). Wide-band tracheids are believed to increase hydraulic adaptability, as they preserve the function of vascular tissues by preventing both cavitation and occlusion during drought-induced shrinking of succulent organs (Landrum, 2006; Mauseth, 2006).

### Water relations

**Fig. 3.**
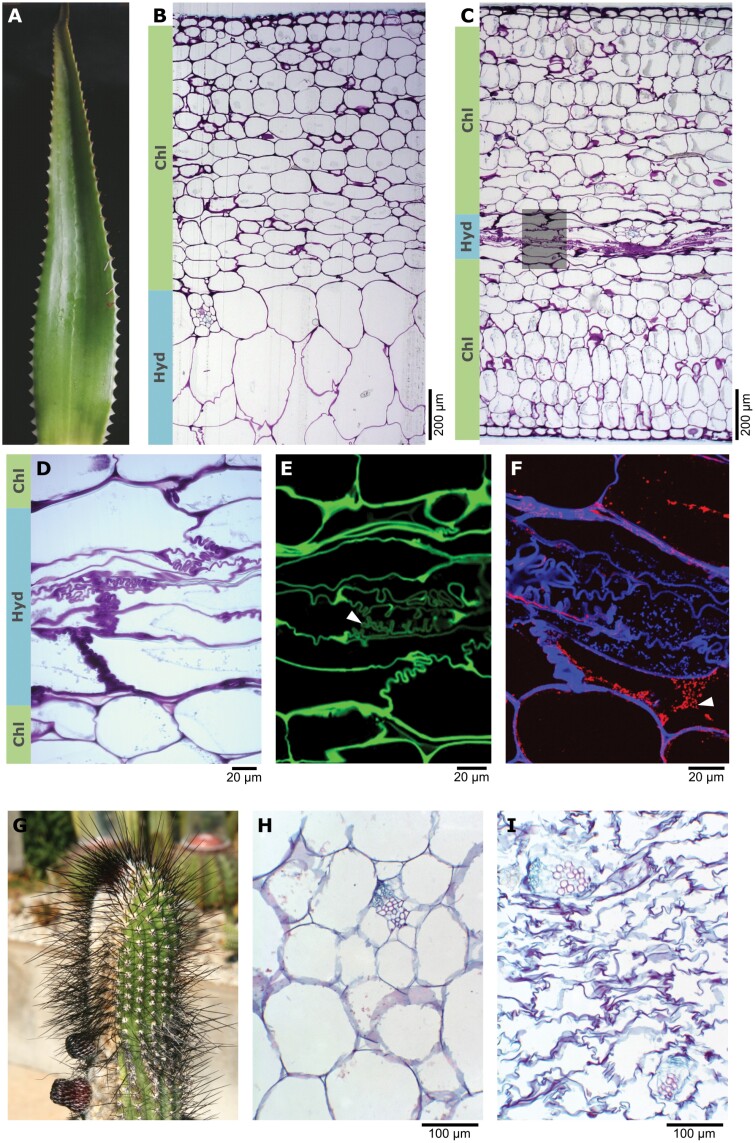
(A–F) Drought response in succulent tissues of *Aloe helenae* (Asphodelaceae). (A) Morphology of a succulent leaf. (B, C) Section of a leaf, stained with toluidine blue, under (B) well-watered and (C) severe drought conditions; note the extreme degree of shrinking of the hydrenchyma upon dehydration. (D) Close-up of the shaded area in (B), showing highly convoluted collapsible cell walls in the hydrenchyma, in contrast to the mostly smooth cell walls in the chlorenchyma. Chl, chlorenchyma; Hyd, hydrenchyma. (E) *In situ* detection of highly de-methyl-esterified HGs using the monoclonal antibody COS^488^ (green signal); note the loss of signal in hydrenchyma cell walls (arrowhead) compared with chlorenchyma. (F) *In situ* detection of acetylated mannans using the monoclonal antibody CCRCM-170 (red signal), with calcofluor white used to stain cellulose in cell walls (blue signal); note the intracellular accumulation of granular mannans (arrowhead). (G–I) Drought response in succulent tissues of *Facheiroa* sp. (Cactaceae). (G) Morphology of a succulent stem of *Facheiroa cephaliomelana* (photo: Pierre Braun; https://commons.wikimedia.org/wiki/File:Facheiroa_tenebrosa_P.J.Braun_%26_Esteves_Bahia_Brasil.jpg; licensed under CC-BY-SA-4.0). (H, I) Stem sections of *Facheiroa ulei* stained with Safranin O/Fast Green FCF of cortex hydrenchyma under (H) well-watered and (I) severe drought conditions. (A–C) Modified from Ahl *et al.* (2019*b*); (H, I) modified from Mauseth (2020).

Unlike non-succulent ‘true’ xerophytes, succulent plants are able to maintain a relatively high water potential (Ψ) even during extended drought (Nobel and Jordan, 1983; von Willert *et al.*, 1992; Griffiths and Males, 2017). Ψ can be calculated according to the simplified formula:


$$\begin{array}{l} \Psi =\Psi_{P}+\Psi_{S} \end{array}$$

where Ψ_P_ is the pressure potential, hydrostatic potential, or turgor pressure, and Ψ_S_ is the solute or osmotic potential (see Taiz *et al.*, 2014). The capacity of succulents to maintain relatively high Ψ is due to high values of hydraulic capacitance (*C*) and low values of volumetric modulus of elasticity (ε) in succulent organs, which is related to highly elastic cell walls (Ogburn and Edwards, 2010). *C* can be defined as:


$$\begin{array}{l} C=\frac{\;\Delta \;V}{\;\Delta \;\Psi \;} \end{array}$$

where Δ*V* is the change in volume, and ΔΨ is the change in Ψ (Nobel, 2009). ε can be defined as:


$$\begin{array}{l} \;\varepsilon \;=\frac{\;\Delta \;{\;\Psi \;}_{P}}{{}^{\;\Delta \;V}╱{}_{V}\;} \end{array}$$

where ΔΨ_P_ is the change in Ψ_P_, and Δ*V*/*V* is the relative volume change; lower values of ε indicate higher elasticity (Nobel, 2009). Cell wall thickness has long been assumed to affect ε (i.e. thicker walls are generally more rigid; Tyree and Jarvis, 1982), and a strong positive correlation has recently been reported (Peguero-Pina *et al.*, 2017). These formulas suggest that cell wall properties influence the trade-offs between maintaining tissue volume and tissue Ψ. The combination of high *C* and low ε means that succulents maintain higher turgor pressure for longer with decreasing Ψ and lose relatively large amounts of water before turgor loss occurs (Bobich and North, 2009; Ogburn and Edwards, 2010). The turgor loss point (TLP_Ψ_; the Ψ at which turgor loss occurs) has generally been interpreted as an indicator of drought tolerance (i.e. tolerating low Ψ) among non-succulent plants (Lenz *et al.*, 2006; Blackman *et al.*, 2010). Many arid-adapted non-succulents respond to drought by lowering their already low TLP_Ψ_ through physiological adjustments, primarily osmotic adjustments (Bartlett *et al.*, 2012; Turner, 2018; Signori-Müller *et al.*, 2021). On the other hand, measurements of TLP_Ψ_ and the closely related Ψ_S_ (see formula in Bartlett *et al.*, 2012) in drought-avoiding succulents have shown that they exhibit relatively high TLP_Ψ_ values (Walter and Stadelmann, 1974; Smith and Lüttge, 1985; von Willert *et al.*, 1992; Donatz and Eller, 1993; Gotsch *et al.*, 2021, Preprint; Leverett *et al.*, 2021); their ability to maintain high Ψ seems to relax the need for a low TLP_Ψ_. Indeed, drought-avoiding succulents are assumed to have a relatively limited capacity for osmotic adjustment (Walter and Stadelmann, 1974; Griffiths and Males, 2017). Given this limitation, if turgor loss is to be prevented during severe, extended drought, elastic adjustment by further decreasing ε may be an important process among drought-avoiding succulents (Schulte, 1992). Such elastic adjustment likely involves rapid changes of the cell wall driven by wall remodelling, particularly of the pectin fraction (Peaucelle *et al.*, 2011; Bethke *et al.*, 2016; Roig-Oliver *et al.*, 2020*b*, 2021*b*). Indeed, changes in the DM of cell wall HGs have been reported as a response to dehydration in the hydrenchyma of *Aloe* species (Fig. 3E) (Ahl *et al.*, 2019*b*). In succulent organs of storage succulents, cell wall heterogeneity between tissues in terms of wall thickness and elasticity allow for preferential water loss and tissue-to-tissue remobilization. As Ψ decreases during the early stages of drought, water is preferentially lost from the large-celled hydrenchyma, given that hydrenchyma cell walls are thinner and more elastic (i.e. lower ε) than those of the chlorenchyma, and this water can then be remobilized to the chlorenchyma to maintain photosynthesis (Schmidt and Kaiser, 1987; Goldstein *et al.*, 1991; Nobel, 2006). This remobilization process seems to be driven by minor osmotic adjustments primarily involving the polymerization or depletion of organic solutes, which create an osmotic gradient (ΔΨ_S_) between hydrenchyma and chlorenchyma (Barcikowski and Nobel, 1984; Schulte and Nobel, 1989; Schulte *et al.*, 1989; Nerd and Nobel, 1991; Herrera *et al.*, 2000).

Despite adaptations of the vascular system to optimize hydraulic connectivity (e.g. Mauseth, 2006; Ogburn and Edwards, 2013; Melo-de-Pinna *et al.*, 2016), succulent organs are generally assumed to have reduced hydraulic conductance [*K*; calculated as *K*_tissue/organ_ = (*K*_X_^–1^ + *K*_OX_^–1^)^–1^; see Sack and Scoffoni, 2013], with outside-xylem hydraulic conductance (*K*_OX_) expected to be particularly limiting due to long outside-xylem hydraulic pathways (Brodribb *et al.*, 2007; de Boer *et al.*, 2012; Ferrio *et al.*, 2012; Sack and Scoffoni, 2013). Water movement in succulents is tightly controlled: emptying of succulent tissues during drought is remarkably slow, whereas refilling upon rain events can happen strikingly quickly (Gibson and Nobel, 1986; Smith and Nobel, 1986; Flach *et al.*, 1995). In transpiring non-succulent leaves, recent evidence suggests that water flow predominantly follows the apoplastic pathway (Buckley, 2015; Buckley *et al.*, 2015). Assuming that the dominance of the apoplastic pathway can be extrapolated to other photosynthetic organs, such as succulent leaves and stems, cell wall features such as thickness, effective porosity, and cell-to-cell connectivity are expected to be among the strongest determinants of *K*_OX_ (Buckley, 2015; Buckley *et al.*, 2015; Bidhendi and Geitmann, 2016; Xiong *et al.*, 2017). Since such features can be modulated through cell wall remodelling, water movement in succulents is likely controlled, at least partially, by cell wall modifications. Among these modifications, pectin remodelling has been postulated as the strongest contributor: conformational changes of pectin due to different enzymatic activities can affect cell wall porosity (McKenna *et al.*, 2010; Levesque-Tremblay *et al.*, 2015; Bidhendi and Geitmann, 2016), and increased cell wall pectin content has been linked to lower cell wall thickness and higher elasticity and hydration (Roig-Oliver et al., 2020*a*, *b*, 2021*a*; Carriquí *et al.*, 2020). Other factors, such as pH and ion concentration, also influence cell wall thickness and extensibility (Demarty *et al.*, 1984; Cosgrove, 2005).

Although the largest reservoir of water in succulent tissues is symplastic, apoplastic water contributes to stored water in some succulent groups, most notably in suborder Portulacineae (Nyffeler, 2007), and is facilitated by a matrix of highly hydrophilic apoplastic polysaccharides known as mucilage (Nobel *et al.*, 1992; von Willert *et al.*, 1992; Ogburn and Edwards, 2010). The term mucilage has also been used interchangeably (and arguably mistakenly) to refer to all water-extractable polysaccharides from succulent tissues (e.g. Sáenz *et al.*, 2004; Ni *et al.*, 2004*a*). Mucilage has been extensively reported in seeds and/or fruits of numerous land plant lineages, which in many cases has also been linked to water retention (Phan and Burton, 2018). Mucilage in succulents occurs in the apoplastic space, either partially filling the space between cells or within the wall of specialized mucilage cells (Nobel *et al.*, 1992; Mauseth, 2006). Mucilage in Cactaceae has been extensively studied and its composition resembles that of pectins, particularly RG-I, with a highly branched structure rich in arabinose and galactose (Cárdenas *et al.*, 1997; Goycoolea and Cárdenas, 2003). Mucilage has also been reported in succulent species of Aizoaceae, Anacampserotaceae, Crassulaceae, Didiereaceae, Portulacaceae, and Vitaceae (Landrum, 2002; Mauseth, 2004*a*), although its role and composition remain unclear.

### Photosynthesis

A recent review by Flexas *et al.* (2021) has highlighted the often-neglected effect of cell wall properties on limiting internal conductance to CO_2_ (*g*_i_) and, thus, on photosynthesis, in addition to the limitation they impose on *K*_OX_. Several interrelated cell wall properties, such as thickness, ε, and effective porosity, have been postulated as some of the strongest determinants of *g*_i_ (Evans *et al.*, 2009; Tosens *et al.*, 2012; Ellsworth *et al.*, 2018; Nadal *et al.*, 2018). However, the influence of cell wall composition on *g*_i_ is still scarcely understood, as indicated by contrasting findings regarding the relationship between pectin content and *g*_i_ (Clemente-Moreno *et al.*, 2019; Carriquí *et al.*, 2020; Roig-Oliver *et al.*, 2020*a*, 2021*a*, *b*). Correlations between *g*_i_ and *K* and their relationship with cell wall parameters indicate coordination between these two parameters and demonstrate the shared cell wall pathway for CO_2_ and water (Flexas *et al.*, 2013; Xiong *et al.*, 2017; Xiong and Nadal, 2020; Roig-Oliver *et al.*, 2021*a*). Throughout land plant evolution, both *g*_i_ and *K* have generally increased with enhanced photosynthetic capacity (de Boer *et al.*, 2012; Flexas and Carriquí, 2020), and such increases have likely been facilitated by changes in cell wall characteristics such as thickness and ε (Nadal *et al.*, 2018; Gago *et al.*, 2019; Carriquí *et al.*, 2020). Thin cell walls and a peripheral distribution of chloroplasts against the cell membrane in succulent tissues (Gibson and Nobel, 1986; von Willert *et al.*, 1992) would suggest that in succulents the cell wall poses a relatively low limitation on *g*_i_ (Evans *et al.*, 2009; Gago *et al.*, 2019; Flexas *et al.*, 2021). However, contrary to the aforementioned evolutionary trend, CAM-performing succulent plants have regressed to states of relatively low *g*_i_, with values being as low as those in gymnosperms, which is thought to increase CAM capacity by limiting internal CO_2_ efflux (Maxwell *et al.*, 1997; Flexas *et al.*, 2008; Ripley *et al.*, 2013). Even though such low *g*_i_ has been previously attributed primarily to anatomical features related to intercellular air spaces (Nelson *et al.*, 2005; Nelson and Sage, 2008), the role of cell wall characteristics in limiting *g*_i_ in succulents remains unexplored.

## Cell walls of succulent tissues under drought

Succulent tissues are characterized by having thin and highly flexible primary cell walls, yet little is known of the mechanism that translates into drought avoidance. Early academic works on succulent tissues noted that distinctive cell wall folding patterns could be observed as cells shrink during drought (Westermaier, 1884; Haberlandt, 1904; Engmann, 1934). Since those early studies, these collapsible cell walls have been reported for a few succulent taxa and are often assumed to be a general anatomical feature of succulents, allowing for controlled regular wall folding and reversible volume changes in succulent organs (Fig. 3). Studies on the cortex hydrenchyma in stems of Cactaceae (Mauseth, 1995) and the hydrenchyma in leaves of *Aloe* (Ahl *et al.*, 2019*b*) have given the most detailed descriptions to date of collapsible cell walls in succulents. This type of cell wall has also been reported in succulent stems of *Euphorbia* (Euphorbiaceae) and Asclepiadoideae (Apocynaceae; Mauseth, 2004*b*), and in succulent leaves of *Sansevieria* (Koller and Rost, 1988*a*, *b*) and *Pyrrosia* (Polypodiaceae; Ong *et al.*, 1992). Although the presence of collapsible cell walls has not been systematically surveyed, histological images from an even broader body of research suggests that collapsible cell walls occur in many more succulent lineages: folding patterns can be observed in succulent tissues of Aizoaceae (e.g. Melo-de-Pinna *et al.*, 2014; Ogura *et al.*, 2018), Crassulaceae (e.g. Jiménez *et al.*, 1983; Sandoval-Zapotitla *et al.*, 2019), Bromeliaceae (e.g. Gomes-da-Silva *et al.*, 2012; Reinert *et al.*, 2013), Gesneriaceae (e.g. Pereira-Dias and Santos, 2015), and Piperaceae (e.g. Horner *et al.*, 2017). When cells in non-succulent plants reach the TLP_Ψ_ under severe drought, negative turgor pressures can develop and result in dehydration injury due to plasmolysis and/or collapse of the cell walls around the plasmolysed protoplasms (Ristic and Cass, 1991; Palomäki *et al.*, 1994; Ding *et al.*, 2014; Vollenweider *et al.*, 2016). On the other hand, succulents maintain relatively high cell turgor pressures and rarely reach the TLP_Ψ_, even during extended drought. As cells in succulent tissues shrink, the convoluted regular folding of collapsible cell walls, coupled with the maintenance of high turgor, points towards a coordinated response that preserves the cell membrane–cell wall continuum and prevents irreversible damage due to mechanical stress. Similarly, cell wall folding in resurrection plants (see Box 1) is thought to prevent the development of negative turgor and subsequent irreversible damage (Oliver *et al.*, 2020; Vander Willigen *et al.*, 2001).

Besides the cell wall and its polysaccharidic components, plant cells also contain carbohydrates within the symplastic domain; all carbohydrates in a tissue, an organ, or a whole plant can be referred to as the glycome. The glycome of some economically important succulent groups has received particular attention due to its multiple applications in pharmaceutics, food, cosmetics, bioremediation, bioenergy, and material sciences (Borland *et al.*, 2009; Grace, 2019). Studies have therefore focused on taxa such as *Aloe* (e.g. Reynolds and Dweck, 1999; Ni *et al.*, 2004*a*), *Opuntia* (Cactaceae; e.g. Goycoolea and Cárdenas, 2003; Ginestra *et al.*, 2009), and *Agave* (e.g. Li *et al.*, 2014; Jones *et al.*, 2020). The interest in *Aloe vera* (L.) Burm.f. and its relatives in Asphodelaceae due to their widespread medicinal uses has fostered one of the most detailed cell wall characterizations in succulent tissues. In the leaf hydrenchyma of *A. vera*, besides structural cell wall polysaccharides, cell contents are rich in storage polysaccharides and free sugars, including the prized acetylated glucomannans, which have putative medicinal properties (Reynolds and Dweck, 1999; Ni *et al.*, 2004*a*, *b*). Subsequent studies have shown that monosaccharide profiles of the hydrenchyma across *Aloe* species and their relatives are phylogenetically constrained, and that well-developed hydrenchyma is the main predictor for medicinal use (Grace et al., 2013, 2015). More recent studies have highlighted the usefulness of high-throughput polysaccharide screening methods such as comprehensive microarray polymer profiling (CoMPP) to characterize the glycomic profiles of succulent tissues (Ahl *et al.*, 2018). Among four *Aloe* species, such profiles exhibited abundant mannans and were shown to vary seasonally (Ahl *et al.*, 2019*a*), which suggests that acclimation processes affecting storage polysaccharides and/or cell walls occur in response to seasonal changes.

Another study on two species of *Aloe* (*A. helenae* and *A. vera*) has confirmed the existence of a tightly regulated cell wall folding process during dehydration (Ahl *et al.*, 2019*b*). Drought-induced pectin remodelling of hydrenchyma cell walls in these *Aloe* species is thought to cause the loss of low-DM HG (Fig. 3E) that is believed to enhance cell wall elasticity and initiate the cell wall folding process. Remarkably, the same study also reported changes in cell wall mannans, including (galacto)(gluco)mannans and acetylated glucomannans, which accumulated inside the cells upon drought in a granular form that resembles that of starch (Fig. 3F). Granular forms of mannans have also been observed in storage organs of *Dendrobium* (Orchidaceae; He *et al.*, 2017) and *Amorphophallus* (Araceae; Ohtsuki, 1968; Chua *et al.*, 2013). The presence of cell wall mannans in the hydrenchyma of *Aloe* was shown to decrease sharply during drought, whereas intracellular mannans increased in the chlorenchyma (Ahl *et al.*, 2019*b*). It has been postulated that, despite not being directly involved in the folding process, mannans in *Aloe* could be acting as CWSPs (see Box 3) by providing energy storage, particularly during drought periods with stalled photosynthesis, and by helping to maintain an osmotic gradient between hydrenchyma and chlorenchyma (Ahl *et al.*, 2019*b*). Mannan mobilization from storage organs has also been reported in orchids and geophytes, and it has been linked to certain growth stages and to the drought stress response by establishing osmotic gradients and promoting water transfer between tissues (Stancato *et al.*, 2001; Tan *et al.*, 2007; Wang *et al.*, 2008; Chua *et al.*, 2013). The reason why *Aloe* and perhaps other succulents seem to rely on mannans as storage during drought, rather than the more widespread starch, probably stems from their different physicochemical properties: starch granules are highly packed and insoluble, and thus exhibit extremely low osmotic activity, whereas soluble mannans possess high osmotic activity and water-holding capacity, and are also mobilized more readily and rapidly than starch (Meier and Reid, 1982; Buckeridge *et al.*, 2000). In storage organs of some orchids and geophytes, the mobilization of mannans occurs before that of coexisting starch (Matsuo and Mizuno, 1974; Franz, 1979), whereas during flowering of *Oncidium* (Orchidaceae) mannans are mobilized from the pseudobulb and subsequently degraded and converted to starch, which temporarily accumulates before further catabolic reactions (Wang *et al.*, 2008). Either way, these observations indicate that mannans can be more easily mobilized than starch, which may be the basis of the use of mannans as CWSPs in *Aloe*.

From different studies, it seems clear that collapsible cell walls in succulents maintain their high elasticity or even increase it further during drought through elastic adjustment, a process that is likely driven by cell wall remodelling (Mauseth, 1995; Ahl *et al.*, 2019*b*). However, the exact mechanism behind this highly regulated process is still largely unknown. Anatomical peculiarities of collapsible cell walls hint at the mechanism behind the folding process: in *Sansevieria* the collapsible walls in the hydrenchyma exhibit bands of secondary thickening (Koller and Rost, 1988*a*, 1988*b*), and it is possible that this ridged spatial patterning of stiffer and softer regions determines how the wall folds. However, most succulent tissues lack secondary wall thickening. Instead, cell wall remodelling can create patterns of local softening and/or loosening and induce phase-separation phenomena in the wall, as seen in many developmental and acclimation processes that require cell growth or a change in cell shape (Peaucelle *et al.*, 2011; Miedes *et al.*, 2013; Amsbury *et al.*, 2016; Bidhendi and Geitmann, 2016; Chebli and Geitmann, 2017; Bidhendi *et al.*, 2019; Haas et al., 2020, 2021). Thus, similar processes leading to localized cell wall softening and/or loosening could be involved in the initiation of the regular cell wall folding process in succulent tissues.

**Fig. 4.**
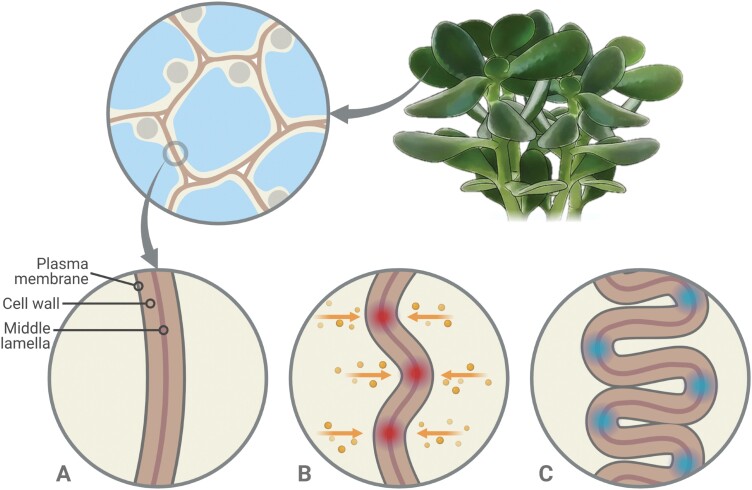
Diagram of the hypothetical cell wall folding process in succulent tissues during drought conditions. (A) Detail of contact region between two cells in a succulent tissue. From a highly hydrated state, initial decreases in relative water content may result in different responses among different succulent lineages: cell wall remodelling may occur in some taxa to increase overall cell wall elasticity and/or to mobilize CWSPs, as seen in *Aloe* (Ahl *et al.*, 2019*b*), whereas other taxa may exhibit constitutively highly elastic cell walls and may not need any modifications at this stage. (B) As relative water content decreases further during extended drought and the cells lose volume, the cell walls experience buckling due to local mechanical stress (in red), which triggers a subcellular response that initiates localized cell wall remodelling (orange arrows). (C) Cell wall remodelling results in patterning of softened and/or loosened regions along the cell wall (in blue), which may act as hinges and facilitate the regular cell wall folding process. Created with BioRender.com.

A hypothetical model, based on the observations of Moore *et al.* (2013) on leaves of resurrection plants, those of Bidhendi *et al.* (2019) on pavement cells of *Arabidopsis*, and those of Ahl *et al.* (2019*b*) on leaves of *Aloe*, is presented in Fig. 4. Cell wall folding can also be observed in plant tissues and organs frequently subjected to desiccation, such as seeds of some plant lineages (Webb and Arnott, 1982) and leaves of some resurrection plants (Cooper and Farrant, 2002; Moore *et al.*, 2006; Oliver *et al.* 2020). In resurrection plants, cell wall folding upon dehydration has been linked to expansin-mediated cell wall loosening, which enhances wall extensibility, and to wall remodelling affecting primarily pectin (Jung *et al.*, 2019), with arabinose-rich polymers (e.g. RG-I arabinans/arabinogalactans and AGPs) postulated as cell wall plasticizers that allow for elastic adjustment (Moore *et al.*, 2013). These cell wall components could act as plasticizers in collapsible cell walls of succulent plants as well. Observations in resurrection plants also suggest that the up-regulation of certain proteins during dehydration-driven cell wall folding, such as glycine-rich proteins (Wang *et al.*, 2009; Giarola *et al.*, 2016) and wall-localized dehydrins (Layton *et al.*, 2010), may help to maintain cell wall integrity and enable repair. As these proteins are ubiquitous among land plants (Sachetto-Martins *et al.*, 2000; Hanin *et al.*, 2011), it is possible that they also play a role in the dehydration response in succulent plants and in regulating the cell wall folding process. However, the high values of cell wall thickness found in resurrection plants makes drawing parallels with drought-avoiding succulents challenging (Flexas *et al.*, 2021; Nadal *et al.*, 2021).

## Future perspectives

The cell wall is a central aspect of drought resistance in plants, yet much remains to be determined about the molecular and physiological mechanisms of cell wall folding processes in drought-avoiding succulents. Cell wall folding in resurrection plants, which has received special attention over the past decades, relies on different mechanisms in different lineages, most of which involve arabinose-rich polymers acting as cell wall plasticizers (Moore *et al.*, 2013). More research is thus needed to elucidate how cell wall folding is regulated in the numerous succulent lineages and whether a shared mechanism exists. In *Aloe*, for instance, it has recently been postulated that HGs and mannans are involved in the folding process (Ahl *et al.*, 2019*b*). Changes in the DM of HGs reinforce the idea that cell wall elasticity is optimized during wall folding, whereas the involvement of mannans suggests that CWSPs and soluble sugars likely play a crucial role during dehydration. Whether similar processes occur in other succulent lineages and whether other cell wall components are involved in the folding process remain to be explored.

As studies of separate cell wall components tend to overlook the complexity of the cell wall and the interactions between different components, holistic approaches should be favoured for cell wall characterization in succulents. Advancing cell wall analytical methods provide promising prospects, with a growing demand for high-throughput methods for rapid screening and profiling of cell wall components (Persson *et al.*, 2011). Spectroscopic methods have been widely used for cell wall characterization (Bauer, 2012; Mansfield *et al.*, 2012; Pettolino *et al.*, 2012; Gierlinger, 2018; Zhao *et al.*, 2020) in combination with imaging techniques (Zhao *et al.*, 2019; Bidhendi *et al.*, 2020; DeVree *et al.*, 2021; Xu *et al.*, 2021). Recent advances in non-destructive real-time imaging, such as light-sheet fluorescence microscopy (LSFM), could allow us to observe changes in the cell walls of succulent tissues under drought in near-physiological conditions (Grossmann *et al.*, 2018; Ovečka *et al.*, 2018). CoMPP, a method based on the specificity of molecular probes, allows high-throughput screening of numerous cell wall components across a wide range of samples (Moller *et al.*, 2007; Rydahl *et al.*, 2018). CoMPP has recently been used alongside immunolocalization to characterize the cell wall and glycomic composition of several *Aloe* species and relatives and to provide a deeper insight into cell wall dynamics under drought (Ahl *et al.*, 2018, 2019*b*). However, the semi-quantitative nature of CoMPP poses some limitations, and it should usually be employed as a complementary method to quantitative biochemical techniques (Moller *et al.*, 2007; Persson *et al.*, 2011). Another disadvantage of CoMPP is the difficulty of isolating succulent tissues within a succulent organ, which is not feasible in most cases and requires whole organs. The latest technological developments include imaging techniques that allow for three-dimensional visualization of cell wall structure, composition, and connectivity, including serial-sectioning scanning electron microscopy (ssSEM; Oi *et al.*, 2017; Harwood et al., 2020, 2021; Antreich *et al.*, 2021) among other high-resolution microscopy techniques (Zeng *et al.*, 2017; Haas *et al.*, 2020), X-ray microcomputed tomography (X-ray microCT; Théroux-Rancourt *et al.*, 2017; Earles *et al.*, 2018), and magnetic resonance imaging (MRI; Malik *et al.*, 2016; Hesse *et al.*, 2020; Mylo *et al.*, 2021). These methods have the potential to elucidate how succulent tissues are built and to reveal their anatomical complexity from a three-dimensional perspective.

While omics studies have shed light on cell wall-related genes and their respective products (Carpita *et al.*, 2001; Minic *et al.*, 2009; Albenne *et al.*, 2013; Houston *et al.*, 2016), genetic tools and resources to specifically study succulents are still largely missing. Genome sequencing of a few succulent taxa over the past decade (Cai *et al.*, 2015; Ming *et al.*, 2015; Copetti *et al.*, 2017; Yang *et al.*, 2017; Jaiswal *et al.*, 2021) offers the possibility of establishing them as models to study drought resistance and/or CAM performance (Yang *et al.*, 2019). Given that succulence has often been regarded as a prerequisite for CAM, engineering CAM into crops and other economically important plants to enhance their water-use efficiency would probably first require the engineering of succulence (Borland *et al.*, 2014; Yang *et al.*, 2015). Since cell walls are expected to play a central role in succulence, next-generation sequencing can be used for future omics studies to mine candidate genes involved in cell wall remodelling in succulent plants (Egan *et al.*, 2012; Strickler *et al.*, 2012; Gross *et al.*, 2013), which would provide opportunities for ongoing (e.g. Lim *et al.*, 2020) and future efforts of engineering tissue succulence into crops.

## Abbreviations

AGP: Arabinogalactan protein
*C*: hydraulic capacitance
CAM: crassulacean acid metabolism
CoMPP: comprehensive microarray polymer profiling
CWSP: cell wall storage polysaccharide
DM: Degree of methyl-esterification
**ε**: volumetric modulus of elasticity
*g*_i_: internal conductance to CO_2_
HG: homogalacturonan
*K*: hydraulic conductance:
*K*_OX_: Outside-xylem hydraulic conductance
*K*_X_: xylem hydraulic conductance
Ψ: water potential
Ψ_P_: pressure potential, hydrostatic potential, or turgor pressure
Ψ_S_: solute or osmotic potential
RG-I: rhamnogalacturonan I
RG-II: rhamnogalacturonan II
TLP_Ψ_: turgor loss point
